# Annotations

**Published:** 1889-01-12

**Authors:** 


### ANNOTATIONS.

**The "Lancet" and the National Pension Fund.**

IN the *Lancet* of January 5th there appeared a rather energetically-expressed letter on the relations of the *Lancet* to the National Pension Fund for Nurses, written by Mr. E. T. Clifford, the honorary manager of the Fund. Mr. Clifford writes with a feeling of soreness, evidently being under the impression that the *Lancet* has not treated the Fund very fairly. The editors of the *Lancet*, in replying to the letter, repudiate the charge of unfair and unkindly-intentioned criticism. So far as can be gathered from their statements, they have no objection to the Fund in itself, and no serious charges to make against it. Their principal contention, which is the parent of all their minor objections, is that the rates charged to nurses are too high. This is a criticism which the *Lancet* or any other editorial or non-editorial person is clearly at liberty to make, but the value of it must as clearly depend upon the fitness of the person making it. The question, therefore, at issue is really this: Are the editors of the *Lancet*, or any anonymous writers they may employ, better able to decide upon the rates to be charged for annuities than the professional actuary who has devised the rates, and other professional actuaries who have approved them? Putting it another way, it may be asked, Does the *Lancet* believe that Sir Andrew Clark is fitter to treat a medical case than the editor of the *Pall Mall Gazette?* Now this is really the crux of the whole business, and if the *Lancet* wishes sensible people to take any notice of its criticisms it must at least bring forward the names of professional actuaries in support of them whose position in the actuarial world is on a level with that of Mr. George King. But, as a matter of fact, whether the rates are really slightly high or not is a question of no importance, and for this simple but conclusive reason, that the Pension Fund is a mutual fund, and the more there is paid in rates the larger will be the profits arising from the invested funds, all of which profits of every kind go to swell the annuities of the insuring nurses. If, therefore, any given nurse pays £21 where £20 would be sufficient to give her an annuity, the only result will be that instead of having the annuity she enters for she will have a much larger one. This being the case, all criticism which confines itself to the question of rates must be considered more or less of the nature of quibbling.

**"Vicarious Digestion."**

"VICARIOUS" is a term more in use among theologians than among physicians, though since the dawn of modern medicine it has become pretty familiar to the latter. It has a significance which ought to excite suspicion in the frank and honest mind. It is opposed to many ideas which commend themselves to the unsophisticated intellect. There is a good general rule which rewards every man according to his own doings, and punishes each for the misdeeds of which he himself is guilty. The doing of things by proxy may be sometimes necessary, but it can never be a universal substitute for individual action and effort. An American physician recently fell foul of what he calls "vicarious digestion;" that is the eating of foods which are either partially digested or are so prepared as to require much less effort in the digestion than it is natural for the unspoilt stomach to put forth. "Our teeth," says he, "are going because there is no longer need of that vigorous mastication peculiar to an age of crude cookery; and if we persist in carrying vicarious digestion to the extent threatened, the stomach will lose its function, and waste away toward the state of a rudimentary organ. The only way to keep the stomach strong is to force it to perform its legitimate work. Vicarious digestion may become a habit if indulged in for too long a time. The 'papoid' habit may become as customary and as destructive as the opium habit. The tissues will starve if fed on cells that enter over the wall instead of by the appointed portals of vital action. Such nutrition does not stay. . . . Food and drinks, feeding and drinking, would seem to exert a wonderful influence over the habits of thought, the customs and manners of races of men, and their diseases also. By searching we might find that the egotism, conservatism, and tenaciousness of the Englishman are as much the results of his beef and ale, as is his gout. . . . America, ever able to give the world a lesson, contributes rush and dyspepsia as the product of brag and whiskey." It would not be easy to demonstrate the worth of these various propositions; but that they are true in spirit and essence need not be doubted. There is a tendency in these days to the "papoid" habit, as the Americans so expressively call it. The habit is as bad as it is, in many cases, necessary; and to many people, as necessary as it is bad. The real fault lies deeper. Its origin is to be found in over-work, over-eating, over-drinking, want of exercise, fresh air, and sound sleep. It is of no use to try to compel a ruined stomach to digest large quantities of substantial and nutritous food. The thing cannot be done. The "papoid" condition is one of the punishments of physiological transgression and stupidity. The cure of it can only be by a slow and tedious process. Regular habits, moderate work, as much exercise in the open air as strength will allow, a gradual return to natural food, a contented mind, in short brains and self-denial. Men and women are not so intellectually feeble as not to understand the "way of health" but many of them are so ineffably stupid as to refuse to walk in it, although they know it as well as they know the face of the town clock. For such, "pap" is the appropriate and truly contemptible diet; and he who finds "pap" fitted for his stomach, will soon find that "pap" also is the only nourishment which his brains and his moral faculty can endure.

**Coughing in Church: a Defence.**

ACCORDING to the *Daily News*, the Rev. David Dickie, of St. Luke's Parish Church, Glasgow, behaved indiscreetly in his pulpit on Sunday afternoon. In London the weather was of the very worst kind, intensely cold and densely foggy If it were the same in Glasgow, all the citizens who had not lungs of cast iron would feel an almost irresistible inclination to cough. Many of them it appears did cough, and that in St. Luke's Parish Church, to the great annoyance of his high mightiness the Rev. David Dickie. The coughing appears to have continued for some time, and to have reached its maximum during the sermon. Whether the sermon itself had anything to do with it or not, we cannot say. St. Luke's congregation, however, will know, and probably many more Glasgow people. It may be affirmed with confidence that Dickie's fame as a preacher has not yet extended beyond the Tweed, if, indeed, it has reached the western shores of the Clyde. Anyhow, the congregation coughed, and the orator's ire was at length aroused. "I consider it time this coughing should cease," said the preacher. "It would have been far better if you had remained in your beds instead of coming here. It is very aggravating for any minister to stand here and try to preach with such disgraceful coughing going on." What the effect of his reverence's "cough specific" may have been we are not informed. Balaam, no doubt was considerably surprised when his ass spake like a man; probably St. Luke's congregation were as much astonished when their man in the pulpit spake as he did. Now, coughing in church is almost always due to one of two causes. Either the church is insufficiently warmed, or the minister is uncommonly dry. Both these causes may be in operation at the same time, and then, of course, the coughing will be very active. But it is clear that the congregation cannot be blamed in either case. Those in charge of the secularities of the church are often extremely careless or extremely stupid in the matter of heating. This is especially the case in Scotland. The writer knows of an old minister in the Highlands who always preached in his overcoat in the winter, and even then used to shiver with cold. No attempt was made to warm the building, although the people must often have sat in ten or twelve degrees of frost. Finally the minister was advised by a friend to have two railway foot-warmers in the pulpit with him, one on each side. It is easy in most cases for church managers to prevent coughing, by taking care that the church is comfortably warmed at least an hour before service, and is kept warm throughout. The removal of the other cause of coughing, a dry preacher, is a rather more difficult matter.

